# Spatial and Temporal Distribution of PM_2.5_ Pollution in Xi’an City, China

**DOI:** 10.3390/ijerph120606608

**Published:** 2015-06-10

**Authors:** Ping Huang, Jingyuan Zhang, Yuxiang Tang, Lu Liu

**Affiliations:** State Key Laboratory of Explosion Science and Technology, Beijing Institute of Technology, 5 South Zhongguancun Street, Haidian District, Beijing 100081, China; E-Mails: jingyuan_Z@163.com (J.Z.); tangyuxiang256@163.com (Y.T.); liulubit@163.com (L.L.)

**Keywords:** PM_2.5_, spatial and temporal distribution, cluster analysis, wavelet transform

## Abstract

The monitoring data of the 13 stations in Xi’an city for the whole years of 2013 and 2014 was counted and analyzed. Obtaining the spatial and temporal distribution characteristics of PM_2.5_ was the goal. Cluster analysis and the wavelet transform were utilized to discuss the regional distribution characteristics of PM_2.5_ concentration (*ρ*(PM_2.5_)) and the main features of its yearly changes and sudden changes. Additionally, some relevant factors were taken into account to interpret the changes. The results show that *ρ*(PM_2.5_) in Xi’an during 2013 was generally higher than in 2014, it is high in winter and low in summer, and the high PM_2.5_ concentration centers are around the People’s Stadium and Caotan monitoring sites; For the regional PM_2.5_ distribution, the 13 sites can be divided into three categories, in which Textile city is Cluster 1, and High-tech Western is Cluster 2, and Cluster 3 includes the remaining 11 monitoring sites; the coefficient of goodness of the cluster analysis is 0.6761, which indicates that the result is acceptable. As for the yearly change, apart from June and July, the average *ρ*(PM_2.5_) concentration has been above the normal concentration criteria of Chinese National Standard (50 g/m^3^); cloudy weather and low winds are the major meteorological factors leading to the sudden changes of *ρ*(PM_2.5_).

## 1. Introduction

In recent years, the problems concerning the influences of smog on the atmospheric visibility, cloud formation, global climate change and human health have aroused a lot of concerns [1,2,3,4]. Some relevant studies have revealed that, particulate matter (PM) is a significant factor in the production of smog [5,6]. PM can cause remarkable increases in the prevalence in the lung cancers [7], and induce damage to the respiratory and cardiovascular systems [8]. PM_2.5_(aerodynamic diameter smaller than 2.5 microns), due to its small size, is able to penetrate the lower respiratory tract to the alveolar sacs and induce cell damage, which has drawn more attention from scholars [7,9]. While the source of PM_2.5_ can vary, it can be a by-product of energy production processes like the burning of coal and petroleum, natural gas and fossil fuels, as well as cigarette smoking and waste incineration [10]. Since the sources of PM_2.5_ are becoming more and more diverse [3,11,12], its prevention and control is becoming much more difficult than ever before. Nowadays the research about PM, especially about PM_2.5_ is focused on its sources [11,12,13,14,15], components [9,16,17,18,19], and chemical analysis [20,21,22]. Specific studies about the spatial and temporal distribution characteristics of PM_2.5_ are seldom found in China, like the ones by Wei Lia and his team who conducted a study on the distribution of atmospheric PM in rural fields, rural villages and urban areas of northern China [12], and Wang [23] who chose the topic of the spatial and temporal variation of air pollution characteristics in China. Alhough they shed some light on the distribution features of PM, all these studies made contain no information about the distribution of PM_2.5_, nor on the factors that influence PM_2.5_ concentrations, despite the fact that to figure out the distribution features of PM_2.5_ and its attributing factors is of great importance for the control and mitigation of smog pollution in China. Considering the above, in this paper, we conducted studies on the following aspects:

- The distribution features of PM_2.5_, on the scale of time and space; and
- The influential distribution factors, from the prospects of human activity and metrological factors.

Since the Chinese government and its environmental protection department have adopted the Air Quality Index (AQI—it contains the PM_2.5_ concentration index) since 2013 and utilized the Air Pollution Index (API—it contains no information about PM_2.5_) before 2013, thus we can only obtain the monitoring data of PM_2.5_ for two years (2013 and 2014) in some pilot cities in China, like Beijing (six monitoring sites), Shanghai (nine monitoring sites) and Xi’an (13 monitoring sites). In order to make the best use of the reliable and comprehensive data, we definitely choose Xi’an city, with 13 monitoring sites, as the study object city. Moreover, Xi’an city is located in the center of Shanxi province in China, which is between 33.42°–34.45° N and 107.40°–109.49° E, and it is regarded as the transportation hub of northwestern China and southwestern, central, and eastern China. The climate in Xi’an city corresponds to the sub-humid warm temperate continental monsoon climate region, with four distinctive seasons. The research about PM_2.5_ distribution on the space and time scale in Xi’an city is therefore representative to some extent.

## 2. Materials and Methods

### 2.1. Data

**Figure 1:**
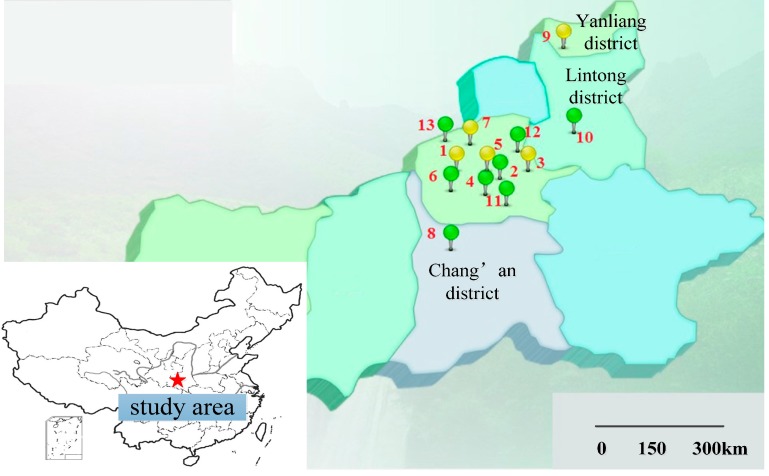
Distribution map of monitoring sites: 1—High-voltage Switch Factory;2—Xingqing district; 3—Textile city; 4—Xiaozhai; 5—People’s Stadium; 6—High-tech Western; 7—Economic development district; 8—Chang’an district; 9—Yanliang district; 10—Lintong district; 11—Qujiang cultural group; 12—Guangyun Tan; 13—Cao Tan.

The distribution map of the 13 monitoring sites is shown in Figure 1, which clearly illustrates the location of the 13 sites. Apart from Chang’an district site, Yanliang district site and Lintong district site, which are relatively scattered, the remaining 10 sites are located centrally. All the sites belong to different regions, and there are some subtle difference in their environmental climates and the industrial structures. These differences can have a significant impact on the monitoring result—the concentration of PM_2.5_. For instance, the High-voltage Switch Factory is located in Lianhu district in northwestern Xi’an city, where the population concentration is high and the northeasterly winds are normal; and the main industry is the production of electro-technical instruments.

The AQI data of Xi’an city from 1 January 2013 to 31 December 2014 has been analyzed statistically. The basic statistical information of the 13 motoring sites and the mean value of the whole city during the two years has been reorganized into Table 1. The original data comes from the website of Xi’an Environmental Monitoring Station, which is a platform site provided by the city government to notify the public about *ρ*(PM2.5Generally, the PM_2.5_ concentration value in 2013 was higher than that in 2014. The standard deviation for all monitoring sites, among which the highest value was 109.554, and the minimum was 63.695, is relatively high, showing that the concentration of PM_2.5_ goes up and down with great fluctuations. The possible reason for this phenomenon is that the concentration is sensitive to all kinds of factors, like temperature and wind velocity, which can cause impressive changes to it. We can learn from the mean value that there are no significant differences in the concentrations of all the monitoring sites.

**Table 1:** Basic statistical information of *ρ*(PM_2.5_) monitoring data in Xi’an city.

Number	Monitoring Sites	Samples		Maximum		Minimum		*ρ*(PM_2.5_)/(μg/m^3^)			
								Mean		Standard Deviation	
		2013	2014	2013	2014	2013	2014	2013	2014	2013	2014
1	High-voltage Switch Factory	365	365	500	500	18	15	144.752	108.156	107.074	71.822
2	Xingqing district	365	365	500	500	13	10	112.621	103.942	105.785	85.320
3	Textile city	365	365	496	470	15	13	129.704	106.948	94.877	68.811
4	Xiaozhai	365	365	500	496	10	10	125.724	97.426	98.628	70.467
5	People’s Stadium	365	365	500	500	18	20	152.915	114.319	109.554	80.374
6	High-tech Western	365	365	500	500	15	5	142.112	99.873	106.453	79.108
7	Economic development district	365	365	474	500	16	15	131.985	102.775	96.902	72.520
8	Chang’an district	365	365	500	430	10	16	122.815	89.431	98.070	66.200
9	Yanliang district	365	365	452	484	15	16	127.734	94.181	92.069	65.968
10	Lintong district	365	365	500	500	13	15	136.406	98.365	102.559	72.012
11	Qujiang cultural group	365	365	500	376	3	12	114.521	95.695	94.720	64.349
12	Guangyun Tan	365	365	500	460	18	18	123.643	95.503	102.280	63.695
13	Cao Tan	365	365	500	500	12	12	163.705	111.151	107.575	80.394

### 2.2. Methodology

The main methods utilized in this paper to discuss the distribution of PM_2.5_ and its influential factors in Xi’an city are cluster analysis and wavelet analysis, and the main tool used was MATLAB-R2012b.

#### 2.2.1. Cluster Analysis

Cluster analysis, which is also called group analysis, is a kind of method that study the classification of samples or index was based on their own features [24]. The so called “cluster”, is a collection of similar elements. The rule of cluster analysis is to compare the features of elements directly, and put the elements with similar qualities into one classification, and elements with different qualities into different classifications. Suppose the number of elements is *n* (13 in this paper), and there are *m* indexes (365 × 2 in this paper) for each element. The basic steps for systematical cluster analysis are the following: firstly, define the distance or similarity coefficient of each element, and the distance of classification and elements are equal. Secondly, put the element (original classification) with shortest distance into a new classification, and calculate the distance between the new classification and other ones. The next step is a repeat operation for the last step, and until all elements are put into one classification, the whole analysis will be stopped. The entire process could be illustrated by the hierarchical clustering map. In this paper, the author intends to develop a deeply data mining based on cluster analysis for the 13 sites, and try to obtain the regional distribution characteristics and its industrial influential factors. The main steps of cluster analysis based on MATLAB-R2012b can be organized as follows:

Step 1: Find the similarities and dissimilarities between different variables in the data set (the monitoring data of 13 sites in this paper), and calculate the distance (Euclidean distance in this paper) through the “pdist” function in MATLAB;
Step 2: Define the linkage between different variables using the “linkage” function;
Step 3: Assess the former calculation effect based on the “cophenetic” function;
Step 4: Establish different clusters through the “cluster” function;
Step 5: Obtain the hierarchial map through “plot”.

#### 2.2.2. Wavelet Transform

Wavelet transformation is new mathematical method which is adopted in all kinds of areas and have been developed rapidly in recent decades. Wavelet transform refers to the use of a limited long or quick attenuated shaking wave (which is called a mother wavelet) to represent a signal [25]. The basic idea of this method is that by decomposing an original signal into a series of primitive signals which contain good frequency domain positioning, and using the characteristics of primitive signals to represent the partial features of the original signal, one can achieve the goal of time and frequency location analysis. The wavelet transform method process includes the establishment of the wavelet function, wavelet transform, and wavelet decomposition and reconfiguration.

The wavelet function is a kind of function with turbulence and quick attenuation characteristics, which is defined as:

$$\int_{-\infty }^{+\infty }\varphi (t)\text{d}t=0$$

In which, φ(t) is also called as primitive wavelet, and its dilation and translation can form a function set:

(1) $$\varphi_{a,b}(t)={\left|a\right|}^{-\frac{1}{2}}\varphi (\frac{b-t}{a}),a\in R,b\in R$$

In Equation (2) $\varphi_{a,b}(t)$ is the branch wavelet, and *a* is the scaling factor which can reflect the cycle, and *b* is time factor, which describes the translation in time. As to the time series $f(t)\in L^{2}(R)$, the branch wavelet in Equation (2) can be transformed as:

(2) $$W_{f}(a,b)={\left|a\right|}^{-\frac{1}{2}}\int_{-\infty }^{+\infty }f(t)\varphi (\frac{b-t}{a})\text{d}t$$

**Figure 2:**
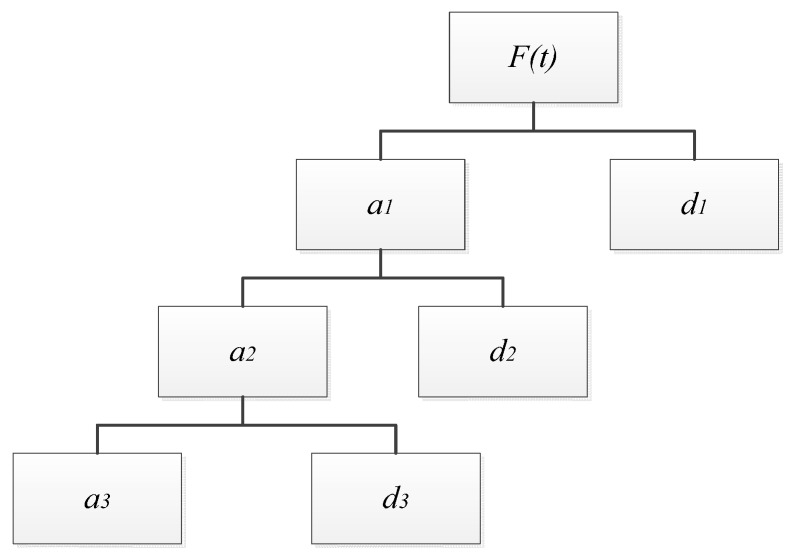
Wavelet decomposition structure.

In Equation (3), $W_{f}(a,b)$ is called a wavelet coefficient, which is the output of f(t) through unit impulse response filter, and it contains the information of *a* and *b*. As for the decomposition of wavelet, it can be described by the tree map shown in Figure 2.

We adopted the “Mallat” algorithm to decompose the original signal (*f(t)*), if the length of the signal is N, then we will obtain the low frequency signal *a_1_* (N/2) and the high frequency signal *d_1_* (N/2) after the level 1 decomposition. After that, we would decompose the low frequency signal *a_1_* in level 2, which would give us the low frequency signal *a_2_* (N/4) and high frequency signal *d_2_* (N/4). Similarly, we will get one low frequency signal and several high frequency signals in the last level decomposition, just as Figure 2 shows. The original signal can be described as the set:

(3) $$F(t)=a_{1}\cup d_{1}\cup d_{2}\cup d_{3}$$

In this process, all the low frequency signals contain the information about the periodic changes, and the high frequency signals contain the information about the sudden changes. Thus, we can utilize the wavelet analysis to figure out the features of *ρ*(PM_2.5_) in both yearly (periodic) change and sudden change.

As for the monitoring data in Xi’an city, if we analyze the original data directly, the yearly change characteristic would be difficult to find because of the huge amount data of 13 sites and 24 months (2013 and 2014); if we adopt the mean value, some specific information would be neglected, like some important sudden change points; similarly, if the Fourier Transform (FT) method of FT is introduced, although the frequency features of PM_2.5_ concentration could be obtained, we cannot get the accurate time corresponding to these frequencies. As a both time and frequency analysis tool, wavelet analysis can provide us some confident results, as assured by relevant studies [20]. Thus, wavelet analysis is a suitable tool to figure out the characteristics of PM_2.5_ concentration in time series.

The Daubechies (db) wavelet, which contains a compactly supported biorthogonal wavelet, presents good performance in time and frequency analysis. In this paper, we utilize the db wavelet to explore the yearly change and sudden change features of PM_2.5_ concentration. The db6 wavelet would be used to find out the yearly change features at level 4 (for its good performance in periodic change analysis), and the db1 wavelet would be adopted to illustrate the sudden feature changes at level 3 (for its impressive effect in sudden change analysis), and the reconstructed coefficient at level 1 and level 2 would be observed to get the sudden change points.

**Figure 3:**
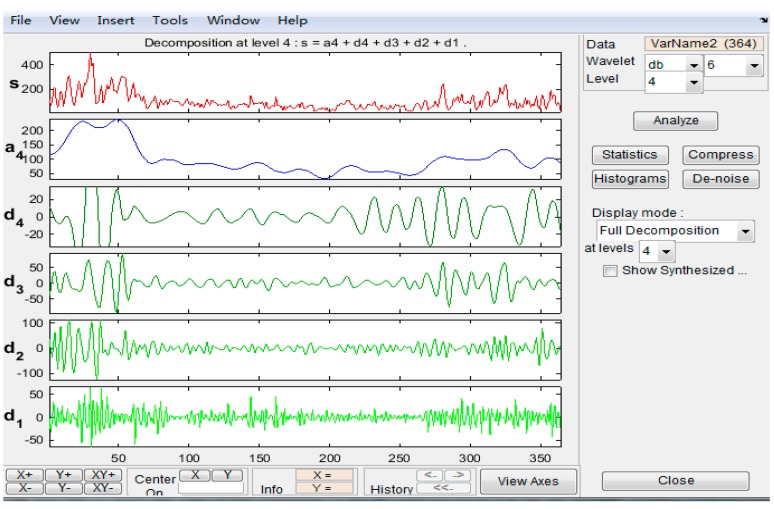
Wavelet analysis platform.

In this paper, we would utilize the “wavelet toolbox” in MATLAB-R2012b to conduct the analysis. Figure 3 shows the analysis platform for the yearly change using the db6 wavelet in Level 4, where we can choose different kind of wavelets in different levels, to obtain different results.

## 3. Results and Discussions

### 3.1. Distribution of ρ(PM_2.5_) in Time and Space

**Figure 4:**
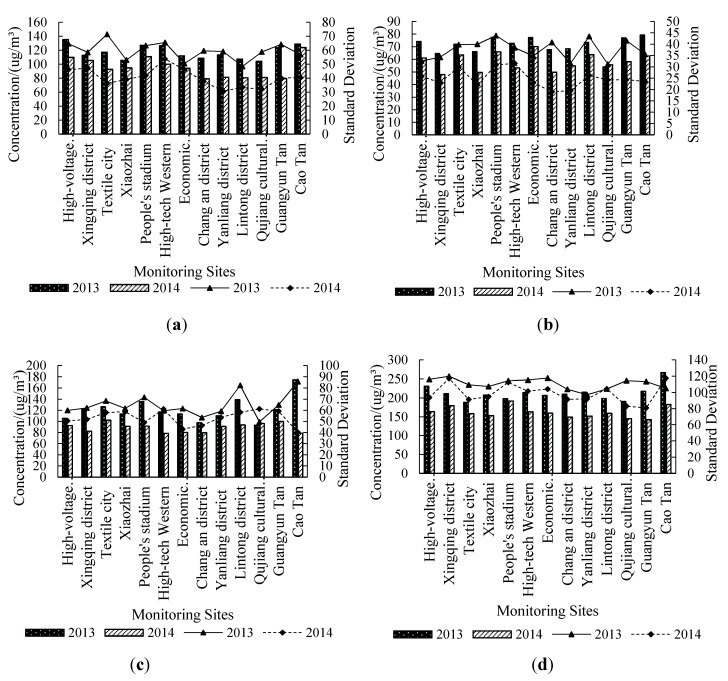
Seasonal distribution of ρ(PM_2.5_) in Xi’an city. (**a**) Spring; (**b**) Summer; (**c**) Autumn; (**d**) Winter.

According to seasonal and the climatic characteristics in Xi’an city, each season should be arranged as follows: Spring contains March, April, and May; Summer holds June, July, and August, Autumn takes September to November; and Winter lasts from December to February in the next year. The distribution situation of PM_2.5_ concentrations for the different seasons in Xi’an city is illustrated in Figure 4.

We can learn from the concentrations shown in the Figure 4 that the concentration of all sites in 2013 is higher than that in 2014, which matches the information contained in Table 1. For different seasons, the highest concentration is in Winter, when the specific mean value reaches 260 μg/m^3^; Autumn is the second worst season, with a mean concentration 170 μg/m^3^; and the concentration of PM_2.5_ is the lowest in Summer with a mean value 75 μg/m^3^.

For the statistic standard deviation for the two years, the value of 2013 is obviously higher than that in 2014, showing that the concentration of PM_2.5_ in 2013 is much more turbulent than that in 2014. For the standard deviation in different seasons, the value in Winter which is nearly three times that in Summer, is the highest, and the value in Autumn and Spring are located between those two extremes. This situation is similar to the seasonal distribution of PM_2.5_ concentration.

**Figure 5:**
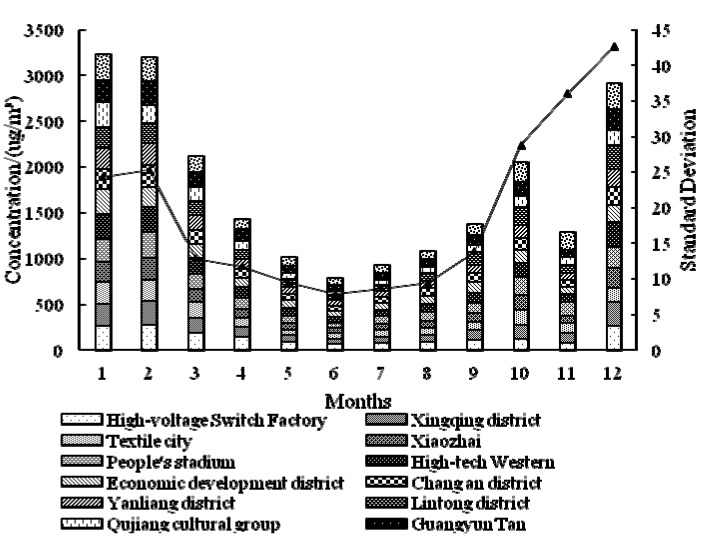
Monthly distribution of *ρ*(PM_2.5_) in Xi’an city for 2013.

**Figure 6:**
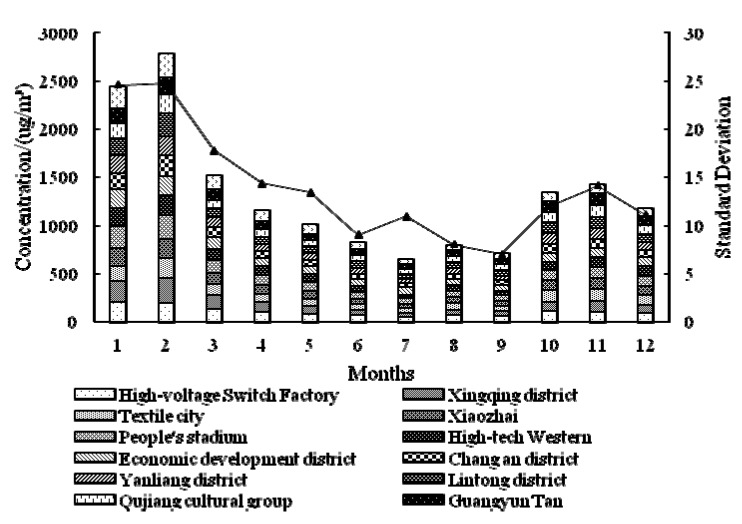
Monthly distribution of *ρ*(PM_2.5_) in Xi’an city for 2014.

In 2013 (as Figure 5 shows), the concentration of PM_2.5_ is higher in January and February than any other months, and the highest center is located in Cao Tan district. The concentration value is continually decreasing in March, April and May, and the highest center is located at the High-voltage Switch Factory in March and April. The concentration in June is the lowest in the whole year, and the mean value for downtown is 60.36 μg/m^3^, which is below the national standard (75 μg/m^3^). The situation in July is similar to that in June, and the mean value is a little bit higher. The concentration value in August, September and October is continually increasing, and the situation of September is definitely different with that in other months—the highest center is located in Lintong district. The spatial distribution of PM_2.5_ is nearly the same for November and December, and the value in December again gets near the highest concentration value. The standard deviation shows that the concentration in June is relatively stable in the whole year. In 2014 (as Figure 6 shows), the overall conditions is similar to those in 2013, but there are several differences: the month of the lowest concentration value is July, and the lowest standard deviation month is September.

### 3.2. Regional Distribution Characteristic of ρ(PM_2.5_)

**Figure 7:**
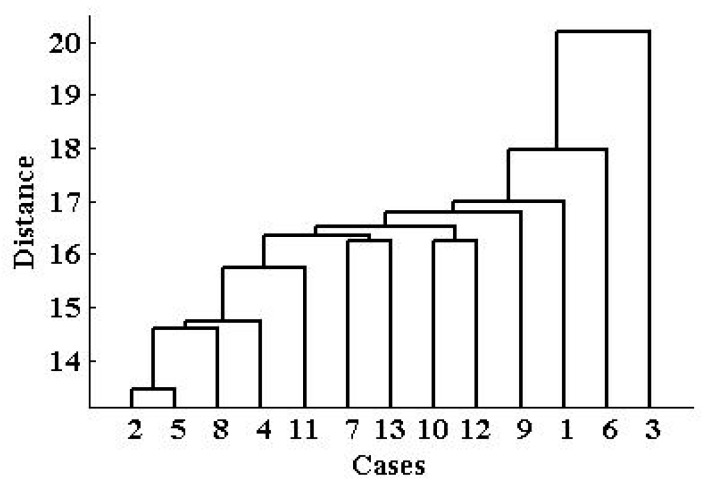
Hierarchical clustering map of 13 monitoring sites in Xi’an city: 1—High-voltage Switch Factory; 2—Xingqing district; 3—Textile city; 4—Xiaozhai; 5—People’s stadium; 6—High-tech Western; 7—Economic development district; 8—Chang’an district; 9—Yanliang district; 10—Lintong district; 11—Qujiang cultural group; 12—Guangyun Tan; 13—Cao Tan.

Cluster analysis is a versatile tool in this study, which can illustrate the inner relationships among behind the monitoring data of the 13 sites. Figure 7 shows the hierarchical clustering map of the 13 sites. Through this map, we can obtain the different clusters, and figure out why the 13 sites could be put into these clusters.

If we define the Euclidean distance is 18, we can get three clusters from the 13 sites based on the PM_2.5_ concentration monitoring data. The reason why we choose 18 as the optimal distance is that, under this condition, the coefficient of goodness of the analysis is 0.6761, which is the best we can get. We can see from the map that Textile city is Cluster 1, and High-tech Western is Cluster 2, and the rest of the sites belong to Cluster 3, which includes the High-voltage Switch Factory district, Xingqing district, Xiaozhai, People’s Stadium, Economic development district, Chang’an district, Yanliang dstrict, Lintong district, Qujiang cultural group, Guangyun Tan, and Cao Tan. Interestingly, we can learn from Figure 1 that, Yanliang district and Lintong district are located far from the other sites, but the cluster analysis similarly puts them into one cluster. Textile city is very near the other sites, but it is put into a cluster alone. This result reveals that the regional distribution of PM_2.5_ is not very related to the geographical locations, which means the climate influence on the regional distribution is not obvious. The most direct impact factor to this result could be the regional industrial production mode and activities. According to the cluster analysis result, we checked out some relevant information, and obtained the main representative industrial activities characteristics of the three clusters, which are collected in Table 2.

We know that Cluster 1 and 2 are often the highest center of *ρ*(PM_2.5_), which is determined by their industrial activities and production structure. For Cluster 3, it is usually not the highest center of *ρ*(PM_2.5_), and the possible reasons for this fact may be that, the proportion of agriculture and tourism in this cluster is quite large. In some specific sites, like Xiaozhai, where the commercial activities are developed, the *ρ*(PM_2.5_) at the site may often be the highest center. However, the specific degree of industrial impacts on *ρ*(PM_2.5_) regional clusters still needs further study.

**Table 2:** The characteristics of the regional industrial activities in Xi’an city.

Cluster	Sites	Main industrial Activities Characteristics
1	Textile city	Dense urban road networks, shopping malls and supermarkets dotted, textiles and clothing, aerospace technology, new materials, equipment manufacturing, modern service industry are the leading production
2	High-tech Western	Electrical machinery, refrigeration equipment, petroleum equipment, instrumentation
3	Xingqing district, Xiaozhai, People’s Stadium, Economic development district, Chang’an district, Yanliang dstrict, Lintong district, Qujiang cultural group, Guangyun Tan, and Cao Tan	Aluminum plant, profile plant, equipment factory, auto parts factory, aircraft industry, paper, flour, commercial vehicle industry, photovoltaic industry, agriculture, tourism

### 3.3. Time Series Analysis of PM_2.5_ Concentration

**Figure 8:**
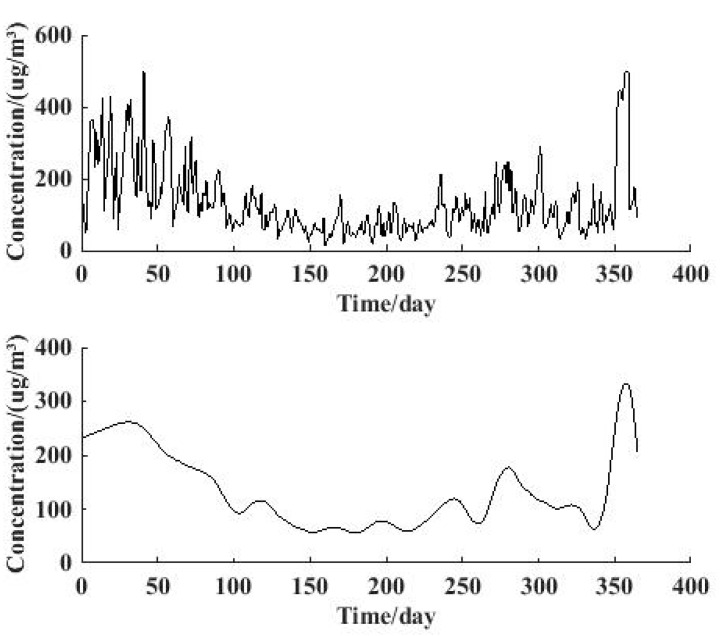
Original signal of *ρ*(PM_2.5_) and 4th level reconstructed low-frequency signal for 2013.

**Figure 9:**
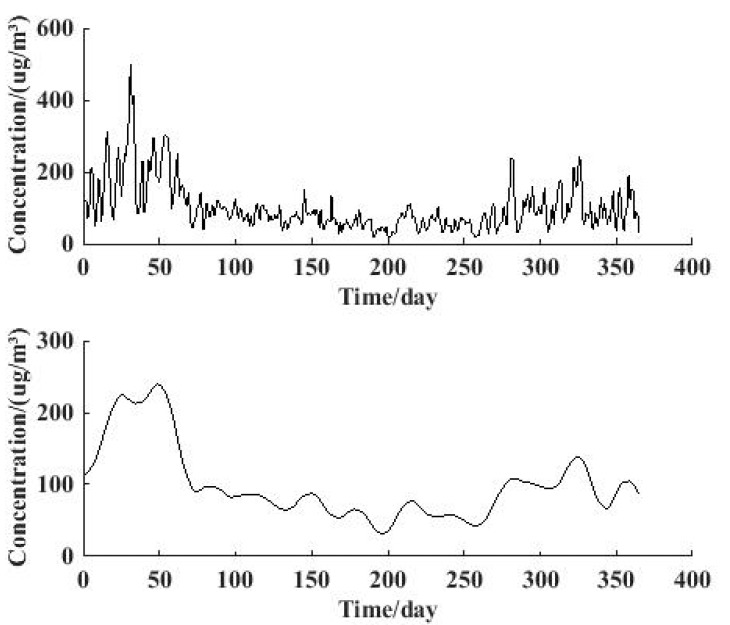
Original signal of *ρ*(PM_2.5_) and 4th level reconstructed low-frequency signal for 2014.

Wavelet analysis is regarded as an excellent method for time series analysis. Figure 8 and Figure 9 describe the result of the wavelet analysis through db6 on the 4th level, and the low frequency signal result contains the yearly change characteristics.

The original PM_2.5_ concentration signal in 2013 is shown in Figure 8(a), and the reconstructed low-frequency signal is illustrated in Figure 8(b). The yearly change characteristics can be obtained directly from the curve: The highest concentration months are January, February and December, and the lowest month is June. The change trend and tendency of PM_2.5_ concentration in the whole year is shown very clearly in Figure 8. Also, the extent of turbulence of the change is contained in the figure, in which the extent of turbulence from April to July is relatively small.

The situation for 2014 is revealed in Figure 9, where we can learn that the overall yearly change characteristics for 2014 are similar to those for 2013. However, the highest concentration months in 2014 are January and February, which is a little different with the situation in 2013. This result is consistent with the information revealed in Figure 3 and Figure 4, which indicates that wavelet analysis is applicable and suitable for studying yearly change of PM_2.5_ concentration.

**Figure 10:**
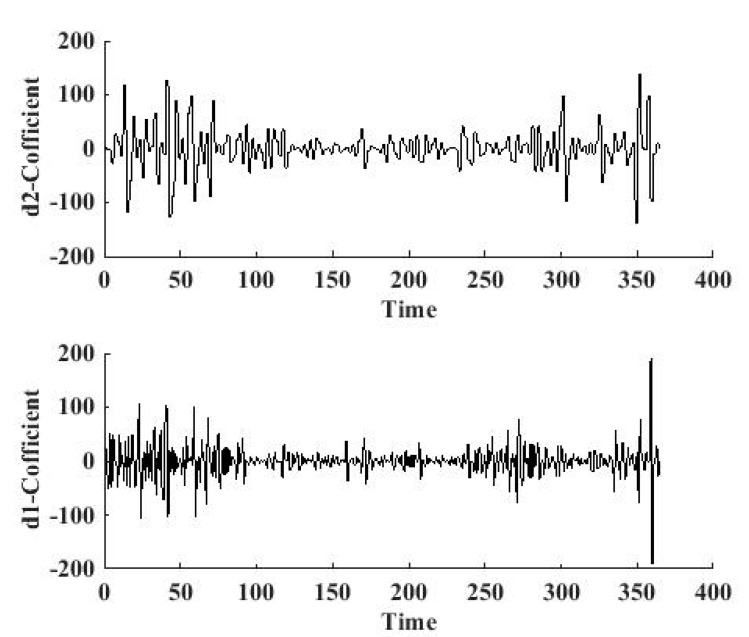
1st level and 2nd level high-frequency signals for 2013.

**Figure 11:**
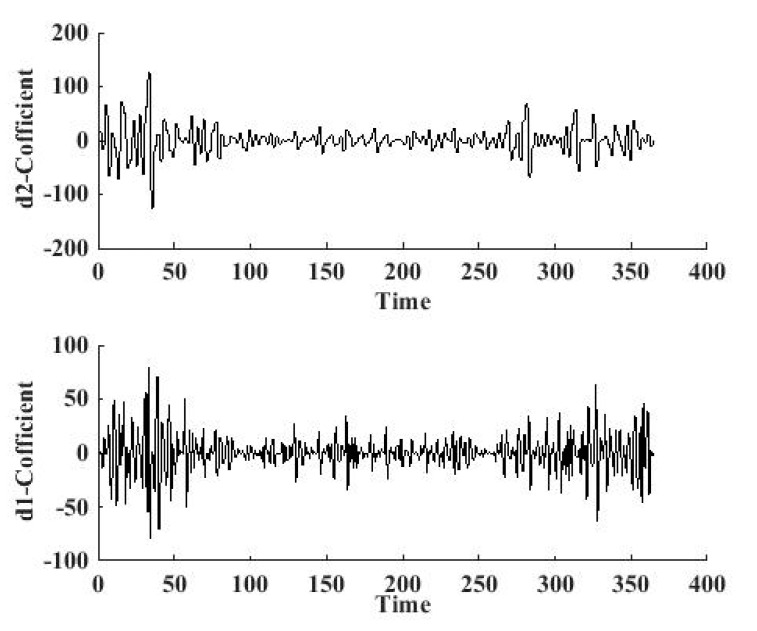
1st level and 2nd level high-frequency signal for 2014.

The sudden changes for 2013 and 2014 are illustrated in Figure 10 and Figure 11, respectively, based on the db1 wavelet. The reconstructed signal at the 2nd level is shown in Figure 10(a), and the reconstructed signal on the 1st level is shown in Figure 10(b). We can obtain from the signal curve on the 2nd level that, there are seven points where the amplitude of the reconstructed coefficients are very big, which means the changes are sudden.

We can obtain the specific dates through zooming in the details in the figure—12th, 42nd, 57th, 71st, 300th, 351st, and 358th. The corresponding dates are: 12 January, 11 February, 26 February, 12 March, 27 October, 17 December, and 24 December.

There are two sudden change points in Figure 11, and the dates are 16 January and 2 February. There are lots of factors that can induce sudden *ρ*(PM_2.5_) changes, for instance, fireworks, gas heating, as well as dust explosions, *etc.* Fireworks can cause an instant increase of *ρ*(PM_2.5_) at a single site, but not in the whole city. Similarly, for gas heating, the fired coal can bring about a *ρ*(PM_2.5_) increase in a time period, but not on a single day. Dust explosions are a kind of accident, which happens occasionally. We have searched the historical records in Xi’an city for the two years, finding that no dust explosions happened. For the considerations above, the human induced factors are not considered in this paper, and we focus on the climate factors that may cause the sudden changes in PM_2.5_ concentration, like temperature, wind speed, and barometric pressure.

**Table 3:** Meteorological data of corresponding mutation day.

Date(mm-dd)		ρ(PM_2.5_) (μg/m^3^)	Pollution Degree	Temperature/°C	Weather Condition	Wind Speed
2013	01-12	264	Severe	−3–7	Cloudy	<3
	02-11	292	Severe	−2–5	Sleety	<3
	02-26	372	Severe	5–13	Cloudy	<3
	03-12	260	Severe	7–16	Cloudy	<3
	10-27	243	Severe	10–19	Cloudy	<3
	12-17	278	Severe	9–12	Hazy	<3
	12-24	500	Severe	−2–3	Hazy	<3
2014	1-16	310	Severe	−3–5	Cloudy	<3
	2-2	413	Severe	1–14	Cloudy	<3

For this purpose we obtained the meteorological data for the sudden change points in Table 3. The data comes from the historical climate records system in Xi’an city. The pollution degrees for the nine sudden change points are all severe. The observation of the meteorological data suggests that the impact of temperature on PM_2.5_ concentration is not so obvious, and still needs further study. What we can decide is that, cloudy days and low wind (<3) are the main meteorological factors for the sudden changes of *ρ*(PM_2.5_).

### 3.4. Discussion

We try to explain the distribution in time and space of PM_2.5_ in Xi’an city geographical locations and meteorological features. Xi’an city is located in the center of China, and belongs to the Semi-humid continental monsoon climate region. A dry and dusty Spring may account for the high concentration of PM_2.5_ in Spring; While in Summer, there is lots of rainy weather and windy days, which can increase the sedimentation ability of the air and help the diffusion of PM_2.5_; In Autumn, low temperatures and winds are very common, both of which are not beneficial for the diffusion of PM_2.5_; The fired coal use for heating in Winter may be the leading factor for the high concentration of PM_2.5_ in December.

Several scholars have tried to find out the distribution of PM_2.5_ in China. The distribution characteristics of PM_2.5_ and PM_10_ in Beijing are an example in this case [26], in which the authors find that the order from high to low of monthly mean concentrations of PM_2.5_ in Beijing is April, February, March, and January; while in this study, the order is January, February, March, and April. The reason for this difference may be the different geographical locations and meteorological factors. Studies of spatial distribution of PM_2.5_ are seldom found. Arc GIS was once used to figure out the distribution map of PM_10_ in the national level in China [23]. The advantages of this method is that the concentration contours can be drawn directly on the map, and the disadvantage is that all the layer cannot match very well, which leads to some blank areas. In this paper, the strength of the method is utilizing the data as much as possible, even though the monitoring sites are distant from each other, but we cannot develop the distribution contour on a single city map.

In the exploration of the regional distribution of PM_2.5_, we adopt the cluster analysis method. For the convenience of analysis, we divide the 13 sites in three clusters, and analyze the correlations between the *ρ*(PM_2.5_) and the industrial activities in each cluster. The results show that Cluster 1 and Cluster 2 which with highest population and diverse industrial activities are often the center of highest *ρ*(PM_2.5_).

The characteristic *ρ*(PM_2.5_) yearly changes and sudden changes have been studied by the wavelet transform method, and some relevant factors have been analyzed. The results show that *ρ*(PM_2.5_) is high in Winter and Spring, and low in Summer and Autumn, which shows the credibility of this method. Meteorological factors are considered to explain sudden changes, and cloudy weather and low wind are the main inducements for the change. The wavelet transform had previously been used in the time series analysis of *ρ*(PM_10_) in Xi’an city in 2001 and 2002 [27]. In that study the author pointed out that the reason for the high *ρ*(PM_10_) is the combined effect of dusty weather, city construction (low wind, temperature inversion, *etc.*), and meteorological conditions. The result of this study is similar to the conclusion of PM_10_ distribution, which reveals that wavelet transform is a suitable tool for the analysis. Additionally, since AQI has only been adopted by the Chinese government for a short time period, and the monitoring of PM_2.5_ is not comprehensive both in time and space, it means the knowledge about it lacks depth as relevant materials are rare to find. All these can bring produce limitations in the study. Further studies should perform a regression analysis between *ρ*(PM_2.5_) and temperature, industrial production value, and wind speed, to figure out the specific correlation of these factors with *ρ*(PM_2.5_). If the data is enough, the change cycle in the time aspect of *ρ*(PM_2.5_) could also be studied.

## 4. Conclusions

This study develops a study of the PM_2.5_ concentration monitoring data of Xi’an city in 2013 and 2014, and the main conclusions are drawn as follows:

(a) The temporal distribution characteristic of PM_2.5_ are that the concentration is higher in Winter, then in Autumn, then in Spring, and concentration in Summer is the lowest. The spatial distribution characteristics of PM2.5 are that the highest concentration center is often located in Caotan, High-tech Western, and Textile city.

(b) Cluster analysis reveals that the cluster of PM_2.5_ distribution is not related to the geographical locations; the concentration of PM_2.5_ in different clusters is the result of industrial activities and the proportion of agriculture.

(c) Wavelet transform illustrates that the characteristics of yearly change and sudden change of PM_2.5_ can be obtained by this method, and the main meteorological factors of sudden change are low wind and cloudy weather.

## Supplementary Files

Supplementary File 1

## References

[1] Han L., Zhou W., Li W., Li L. (2014). Impact of urbanization level on urban air quality: A case of fine particles (PM_2.5_) in Chinese cities. Environ. Pollut..

[2] Fu M., Martínez-Sánchez J.M., Galán I., Pérez-Ríos M., Sureda X., López M.J., Schiaffino A., Moncada A., Montes A., Nebot M. (2013). Variability in the correlation between nicotine and PM_2.5_ as airborne markers of second-hand smoke exposure. Environ. Res..

[3] Negral L., Moreno-Grau S., Moreno J., Querol X., Viana M.M., Alastuey A. (2008). Natural and Anthropogenic Contributions to PM_10_ and PM_2.5_ in an Urban Area in the Western Mediterranean Coas. Water Air Soil Pollut..

[4] Galea K.S., Hurley J.F., Cowie H., Shafrir A.L., Sánchez Jiménez A., Semple S., Ayres J.G., Coggins M. (2013). Using PM_2.5_ concentrations to estimate the health burden from solid fuel combustion, with application to Irish and Scottish homes. Environ. Health.

[5] Shu S., Yang P., Zhu Y. (2014). Correlation of noise levels and particulate matter concentrations near two major freeways in Los Angeles, California. Environ. Pollut..

[6] Wang J., Zheng J., Ke Z. (2012). Assessment of PM_2.5_ Exposure of residents in a community downwind of a typical industrial source. Res. Environ. Sci..

[7] Leung P.Y., Wan H.T., Billah M.B., Cao J.J., Ho K.F., Wong C.K. (2014). Chemical and biological characterization of air particulate matter 2.5, collected from five cities in China. Environ. Pollut..

[8] Langrish J.P., Li X., Wang S., Lee M.M., Barnes G.D., Miller M.R., Cassee F.R., Boon N.A., Donaldson K., Li J. (2012). Reducing personal exposure to particulate air pollution improves cardiovascular health in patients with coronary heart disease. Environ. Health Perspect..

[9] Rogge W.F., Ondov J.M., Bernardo-Bricker A., Sevimoglu O. (2011). Baltimore PM_2.5_ Supersite: Highly time-resolved organic compounds—Sampling duration and phase distribution—Implications for health effects studies. Anal. Bioanal. Chem..

[10] Kong S., Ding X., Bai Z., Han B., Chen L., Shi J., Li Z. (2010). A seasonal study of polycyclic aromatic hydrocarbons in PM_2.5_ and PM_2.5–10_ in five typical cities of Liaoning Province, China. J. Hazard. Mater..

[11] Wang H., Zhuang Y., Wang Y., Sun Y., Yuan H., Zhuang G., Hao Z. (2008). Long-term monitoring and source apportionment of PM_2.5_/PM_10_ in Beijing, China. J. Environ. Sci..

[12] Sun Y., Zhou X., Wai K.Y., Yuan Q., Xu Z., Zhou S., Qi Q., Wang W. (2013). Simultaneous measurement of particulate and gaseous pollutants in an urban city in north China plain during the heating period: Implication of source contribution. Atmos. Res..

[13] Xiao Z.-M., Bi X.-H., Feng Y.-C., Wang Y.-Q., Zhou J., Fu X.-Q., Weng Y.-B. (2012). Source apportionment of ambient PM_10_ and PM_2.5_ in urban area of Ningbo city. Res. Environ. Sci..

[14] Kim H.S., Chung Y.S., Lee S.G. (2012). Characteristics of aerosol types during large-scale transport of air pollution over the Yellow Sea region and at Cheongwon, Korea, in 2008. Environ. Monit. Assess..

[15] Callén M.S., Iturmendi A., López J.M. (2014). Source apportionment of atmospheric PM_2.5_-bound polycyclic aromatic hydrocarbons by a PMF receptor model. Assessment of potential risk for human health. Environ. Pollut..

[16] Hernández-Mena L., Murillo-Tovar M., Ramírez-Muñíz M., Colunga-Urbina E., de la Garza-Rodríguez I., Saldarriaga-Noreña H. (2011). Enrichment factor and profiles of elemental composition of PM_2.5_ in the city of Guadalajara, Mexico. Bull. Environ. Contam. Toxicol..

[17] Mkoma S.L., da Rocha G.O., Regis A.C.D., Domingos J.S.S., Santos J.V.S., de Andrade S.J., Carvalho L.S., de Andrade J.B. (2014). Major ions in PM_2.5_ and PM_10_ released from buses: The use of diesel/biodiesel fuels under real conditions. Fuel.

[18] Pastuszka J.S., Rogula-Kozłowska W., Zajusz-Zubek E. (2010). Characterization of PM_10_ and PM_2.5_ and associated heavy metals at the crossroads and urban background site in Zabrze, upper Silesia, Poland, during the smog episodes. Environ. Monit. Assess..

[19] Qian J., Han J., Ruan X. (2014). Analysis of the high resolution variation of PM_2.5_ and its carbonacious components at Xi’an during high pollution period in winter. Ecol. Environ. Sci..

[20] Ma J., Chen Z., Wu M., Feng J., Horii Y., Ohura T., Kannan K. (2013). Airborne PM_2.5_/PM_10_-associated chlorinated polycyclic aromatic hydrocarbons and their parent compounds in a suburban area in Shanghai, China. Environ. Sci. Technol..

[21] Vicente A.B., Pallares S., Soriano A., Sanfeliu T., Jordan M.M. (2011). Toxic Metals (As, Cd, Ni and Pb) and PM_2.5_ in Air Concentration of a Model Ceramic Cluster. Water Air Soil Pollut..

[22] Li W., Wang C., Wang H., Chen J., Yuan C., Li T., Wang W., Shen H., Huang Y., Wang R. (2014). Distribution of atmospheric particulate matter (PM) in rural field, rural village and urban areas of northern China. Environ. Pollut..

[23] Wang B. (2008). The Spatial and Temporal Variation of Air Pollution Characteristics in China Adopting Air Pollution Index (API) Analysis.

[24] Gao H.X., Qiu S. (2005). Cluster Analysis. Applied Multiple Statistical Analysis.

[25] Liu G., Di S. (2001). Wavelet Analysis and Its Application.

[26] Yu J., Yu T., Wei Q., Wang X., Shi J.-G., Li H.-J. (2004). Characteristics of Mass Concentration Variations of PM_10_ and PM_2.5_ in Beijing Area. Res. Environ. Sci..

[27] Chen L., Ma G. (2006). The application of wavelet in the time serious analysis of PM_10_. Environ. Eng..

